# Identification of molecular subtypes and immune infiltration in endometriosis: a novel bioinformatics analysis and *In vitro* validation

**DOI:** 10.3389/fimmu.2023.1130738

**Published:** 2023-08-18

**Authors:** Si-ji Lv, Jia-ni Sun, Lei Gan, Jing Sun

**Affiliations:** ^1^ Shanghai Key Laboratory of Maternal Fetal Medicine, Shanghai Institute of Maternal-Fetal Medicine and Gynecologic Oncology, Shanghai First Maternity and Infant Hospital, School of Medicine, Tongji University, Shanghai, China; ^2^ Department of Gynaecology and Obstetrics, Ningbo First Hospital, Ningbo, Zhejiang, China

**Keywords:** endometriosis, WGCNA, signature, immune infiltration, immune cell subset, molecular subtype

## Abstract

**Introduction:**

Endometriosis is a worldwide gynacological diseases, affecting in 6–10% of women of reproductive age. The aim of this study was to investigate the gene network and potential signatures of immune infiltration in endometriosis.

**Methods:**

The expression profiles of GSE51981, GSE6364, and GSE7305 were obtained from the Gene Expression Omnibus (GEO) database. Core modules and central genes related to immune characteristics were identified using a weighted gene coexpression network analysis. Bioinformatics analysis was performed to identify central genes in immune infiltration. Protein-protein interaction (PPI) network was used to identify the hub genes. We then constructed subtypes of endometriosis samples and calculated their correlation with hub genes. qRTPCR and Western blotting were used to verify our findings.

**Results:**

We identified 10 candidate hub genes (GZMB, PRF1, KIR2DL1, KIR2DL3, KIR3DL1, KIR2DL4, FGB, IGFBP1, RBP4, and PROK1) that were significantly correlated with immune infiltration. Our study established a detailed immune network and systematically elucidated the molecular mechanism underlying endometriosis from the aspect of immune infiltration.

**Discussion:**

Our study provides comprehensive insights into the immunology involved in endometriosis and might contribute to the development of immunotherapy for endometriosis. Furthermore, our study sheds light on the underlying molecular mechanism of endometriosis and might help improve the diagnosis and treatment of this condition.

## 1 Introduction

Endometriosis is a chronic gynecological disorder characterized by the presence and infiltration of ectopic endometrial tissue (1, 2). It affects nearly 5% to 10% of women of reproductive age and could cause pelvic pain (50% to 80%) and infertility (up to 50%) (3–6). Other common symptoms include dysmenorrhea, dyspareunia, dysuria, dyschezia, and fatigue (2, 4). Although various biomarkers such as IL-2, anti-PEP, CA125, and miR-150-5p have been studied as clinical diagnostic tools, there is generally no single indicator that can directly diagnose endometriosis (7). Surgery remains the gold standard for diagnosis (8), but it has complications, and early lesions might be missed during operations (9). Therefore, a thorough understanding of the molecular mechanisms underlying endometriosis and exploration of a non-invasive diagnostic indicator with high specificity is essential.

Sampson’s retrograde stigma theory is the most widely accepted theory on the pathogenesis of endometriosis (6). However, endometriosis is now recognized as a systemic disease that can spread through lymphatic and hematogenous metastases (10), not just limited to pelvic lesions (6). In addition, recent research suggests that endometriosis is closely related to inflammation and autoimmunity (11, 12).

Nowadays, the bioinformatics approach is widely used to reveal the molecular mechanisms associated with endometriosis. The underlying pathogenic factors are variable and could be related to the lesion microenvironment, individual differences, and environmental factors (4). As mentioned earlier, endometriosis is closely related to immunity, and some studies have revealed a part of the mechanisms. For instance, Wu et al. (13) reported that an increase in CD8^+^ T cells and a decrease in CD163^+^ macrophages might create a pro-inflammatory endometrial immune environment, leading to endometriosis. Zhong et al. (14) found that M2 macrophages significantly increase during the progression of endometriosis. In a previous study, it was discovered that PDLIM3, a specific biomarker in endometriosis, was correlated with multiple immune cells, such as M2 macrophages, activated NK cells, and CD8^+^ T cells (15). However, many unknowns still exist regarding the immune mechanisms of endometriosis.

In our study, we downloaded three endometriosis datasets (GSE51981, GSE6364, and GSE7305) from the GEO database to evaluate immune cell infiltration and identify immune correlation with endometriosis for further analysis. We identified endometriosis subtypes, explored differentially expressed genes (DEGs), and constructed a co-expression network. Central genes were identified, and subsequently, hub genes were comprehensively analyzed, with a particular focus on their immunological characteristics. Our study established a detailed immune network and aimed to systematically clarify the underlying molecular mechanisms of endometriosis, particularly from the perspective of immune infiltration. The results of our study could help facilitate the development of immune therapeutic approaches for endometriosis.

## 2 Materials and methods

### 2.1 Data collection and preprocessing

**Figure 1:**
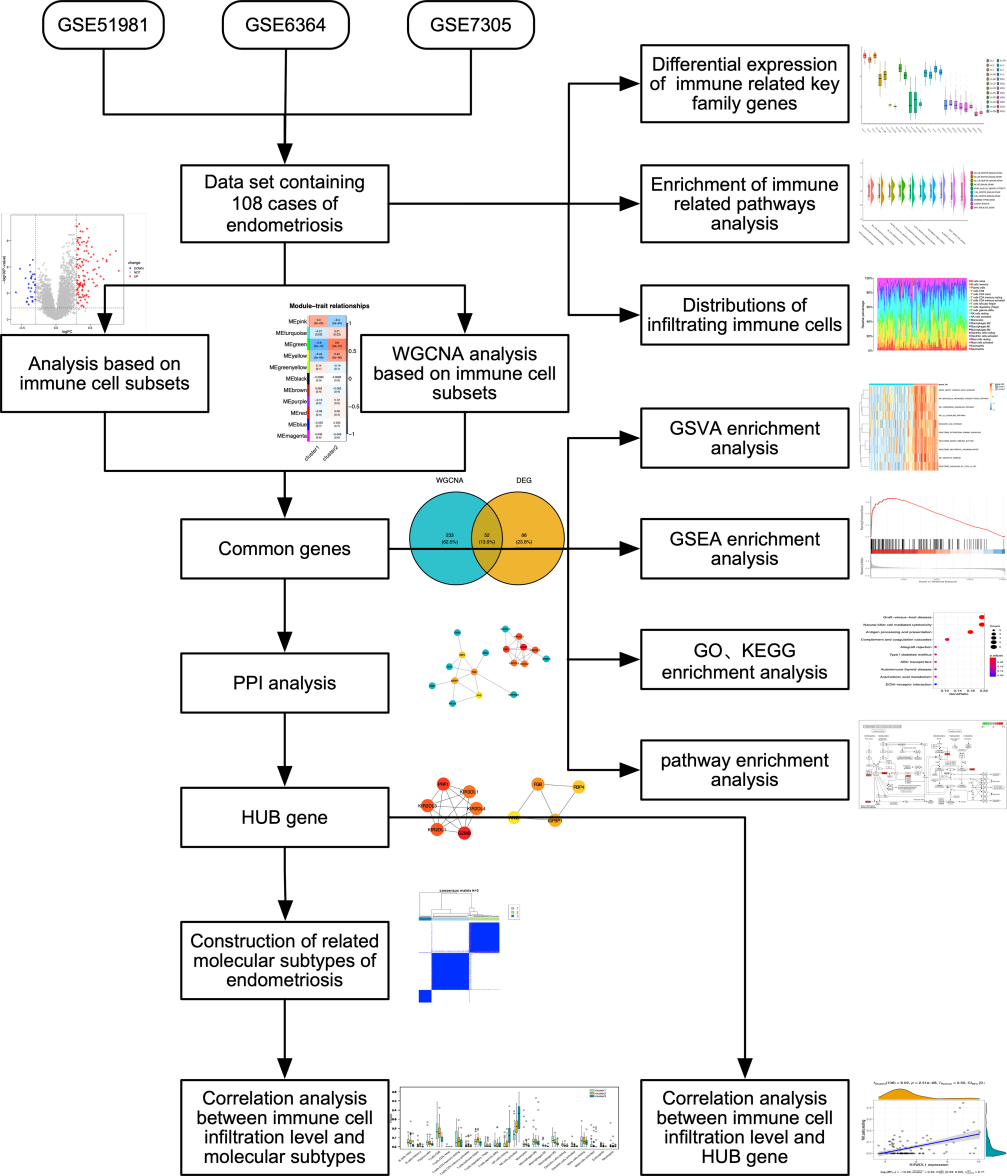
Flow diagram of methodologies applied to explore the biological characteristics of endometriosis.

To identify DEGs, the expression profiles of GSE51981, GSE6364, and GSE7305 were obtained from the GEO database using the GPL570[HG-U133_Plus_2] Affymetrix Human Genome U133 Plus 2.0 Array. We used the GEOquery (16) package of R software with the keyword “endometriosis”. GSE5198 included 77 endometriosis samples, GSE6364 included 21 endometriosis samples, and GSE7305 included 10 endometriosis samples. We selected and combined the endometriosis samples from the three datasets, which underwent removal of batch effects, standardization and annotation of probes, and other data cleaning processes. We used the “removeBatchEffect” function to remove batch effects using the “limma” R package and employed “normalizeBetweenArrays” function to standardize the data. The overall analytical flow diagram is shown in **Figure 1**.

### 2.2 Evaluation and analysis of immune cell infiltration and correlation between immune cells

We used the CIBERSORT algorithm (17) to convert the normalized gene expression matrix into a matrix of 22 types of immune cells. We uploaded the gene expression matrix data to CIBERSORT and filtered out samples with P < 0.05 to obtain the immune cell infiltration matrix. A total of 108 samples were uploaded, and 32 samples were filtered out based on the criteria of P < 0.05. The Ggplot2 (18) package was used to draw bar charts showing the distribution of the 22 immune cell infiltrates in each sample. The Corrplot package was used to plot the correlation heatmap to visualize the correlations of these 22 immune cell infiltrates. The Ggplot2 package was used to visualize the expression of immune-related HLA family and KIR family genes.

### 2.3 Gene set variation analysis

GSVA (19) is a non-parametric, unsupervised analysis that calculates enrichment scores for specific sets of genes in each sample. GSVA contributes to the construction of pathway-central models of biology and meets the need for bioinformatic methods for RNA-seq data (20). In our study, we used the expression profile data of endometriosis patients based on the c2.cp.v7.4.symbols.gmt dataset to analyze the corresponding biological characteristics and observe changes in immune-related pathways to explore the underlying mechanism of pathogenesis in endometriosis. The c2.cp.v7.4.gm geneset is a collection of gene sets in the Molecular Signatures Database (MSigDB) version 7.4. It contains co-expression-based gene sets that capture potential functional relationships among genes across diverse tissues or cell types (https://docs.gsea-msigdb.org/#MSigDB/Release_Notes/MSigDB_7.4/#_top).

### 2.4 Construction and analysis of related molecular subtypes

Consensus clustering involves the identification of molecularly related subtypes using the Consensus Cluster Plus algorithm (21). The consensus matrix is determined by consensus clustering to classify the samples, with the k value of the cluster number set between 2 and 9. When the cumulative distribution function index reaches the approximate maximum value, the optimal K value is determined. The classification is then validated by principal component analysis (PCA) of mRNA expression profiles.

The construction of immune cell-related subtypes was based on the consensus clustering of immune cell infiltration data. The results were used to define Cluster 1 and Cluster 2. DEGs were identified by limma (22) with the criteria of P-value < 0.05 and |log2FC| > 0.3. We used the ggplot2 package to draw a volcano plot and a heatmap of the DEGs to visualize their expression.

### 2.5 Weighted gene co-expression network based on immune cell subsets

We used WGCNA based on immune cell subsets (23) to process data. We used the c2.cp.v7.4.symbols.gmt dataset, which includes immune-related pathways and genes selected from the dataset obtained through GSVA. First, the soft threshold value of network construction was selected, and the adjacency matrix was a continuous value between 0 and 1, ensuring that the constructed network followed a power-law distribution and was closer to the real biological network state. Second, a scale-free network was constructed using the function of block modules, and the co-expression modules were identified by block partitioning analysis, grouping genes with similar expression patterns. All modules were summarized by modular characteristic genes, which were the most important major components of each module and were defined as synthetic genes representing the expression profile of all genes in a given module.

### 2.6 Functional enrichment analysis

The ClusterProfiler package (24) was used for Gene Ontology (GO) and Kyoto Encyclopedia of Genes and Genomes (KEGG) enrichment analysis of the DEGs, and the common genes were analyzed using WGCNA. The critical value of false discovery rate < 0.05 was considered statistically significant. The GO and KEGG results were visualized using the pathview package (25), and the first two KEGG enrichment results were evaluated.

To investigate the differences in biological processes among different subgroups, we used gene set enrichment analysis (GSEA) based on the gene expression profile dataset of patients with endometriosis. The C2.cp.v7.4.symbols.gmt gene set was downloaded from MSIGDB for GSEA, and a P-value < 0.05 was considered statistically significant.

### 2.7 PPI network construction

We used the STRING database (26) (http://string-db.org, version 11.09) online tool to obtain PPI data. Candidate targets were input into STRING, and genes with scores greater than 0.3 were selected to construct a network model using Cytoscape (V3.7.2) (27). Cytohubba plug-in (28) was used to screen the top 10 hub genes based on the score. Pearson correlation analysis was conducted between the hub genes and immune cells, and both cor > 0.55 and > 0.7 were tried. An interaction score of 0.55 and P < 0.05 was finally set as the significance criteria for further analysis. The correlation between hub genes and immune checkpoint genes was assessed as previously using corrgram (29).

### 2.8 Construction of miRNA-mRNA interaction network

The MultiMiR (30) package was used to construct the interaction between mRNA and miRNA. We obtained data from the miRTarBase database (http://mirtarbase.mbc.nctu.edu.tw/php/index.php) and TarBase V.8 database (http://carolina.imis.athena-innovation.gr/diana_tools/web/index.php?r=tarbasev8%2Findex). The “Functional MTI” and “positive” results were selected, and Cytoscape (V3.7.2) was used to construct the miRNA-mRNA network.

### 2.9 Quantitative real-time PCR

Total RNA was isolated from 6 ovarian endometriosis tissue samples and 9 eutopic endometrial tissue samples from endometriosis-free women (normal control, NC) using Trizol (RNAiso Plus; Takara, Japan) according to the manufacturer’s instructions. One microgram of RNA was reverse-transcribed into cDNA using a PrimeScript™ RT reagent kit (Takara) according to the manufacturer’s protocol. Amplification was performed on a quantitative real-time (qRT)-PCR device (QuantStudio5; Thermo Fisher Scientific, Waltham, MA, USA) using TB Green^®^ Premix EX TaqTM II (Takara) and gene-specific primers (Sangon, Shanghai, China). β-Actin was used as the internal control. The relative expression levels of the genes were calculated using the 2^-ΔΔCT^ method.

### 2.10 Protein extraction and western blotting

Ovarian endometriosis tissues and normal eutopic endometrium tissues were lysed in RIPA buffer containing protease and phosphatase inhibitor cocktail. After centrifugation at 12,000 rpm and 4°C for 15 min, the protein concentration in the supernatant was determined using a BCA protein assay. Equal amounts of total protein (30 µg) were loaded and separated on 12.5% SDS-PAGE and then transferred to PVDF membranes. After blocking with protein-free rapid blocking buffer for 0.5 h at room temperature, the membranes were incubated with primary antibodies overnight at 4°C. Rabbit anti-RBP4 antibody (1:5000; Abcam) and rabbit anti-β-actin (1:1000, cell signaling technology) were used as primary antibodies, with β-actin serving as the internal loading control. Then, the membranes were then incubated with secondary antibodies (1:3000) for 1 h at room temperature. Excess secondary antibody was removed by three washes in TBST. The targeted protein bands were visualized and imaged using an ECL Western blotting kit (NCM Biotech, China).

### 2.11 Statistical analysis

All data processing and analysis were conducted using R software (version 4.1.0). The statistical significance of the normally distributed continuous variables was estimated using the independent Student t test, while the differences between non-normally distributed continuous variables were analyzed using the Mann-Whitney U test (Wilcoxon rank-sum test). The Chi-square test or Fisher’s exact test was used to compare and analyze the statistical significance between categorical variables in two groups. All statistical P values were bilateral, and P < 0.05 was considered statistically significant.

## 3 Results

### 3.1 Evaluation of immune cells infiltration and immune correlation analysis in endometriosis

**Figure 2:**
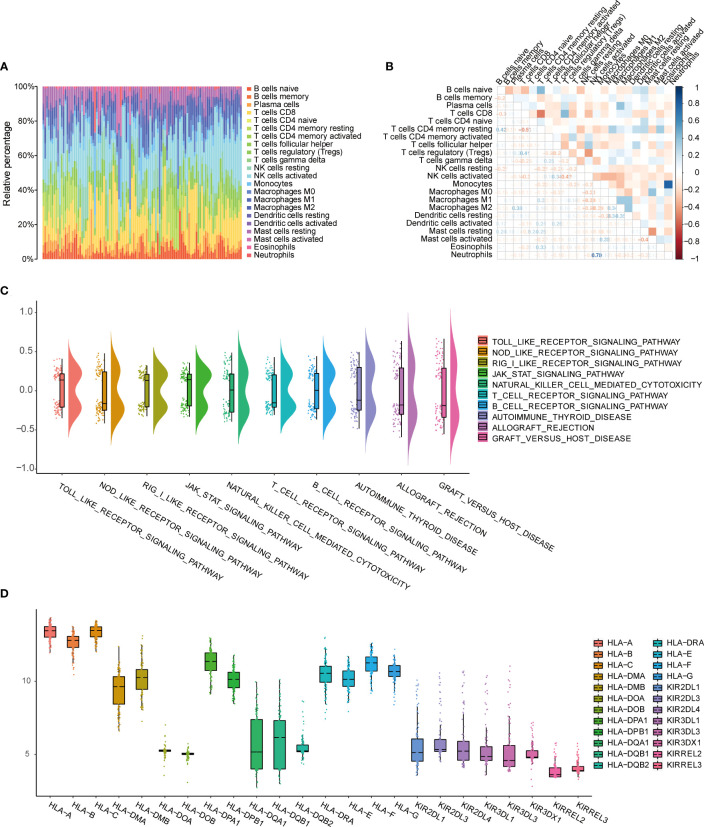
Evaluation of immune cell infiltration and immune correlation analysis. **(A)** Barplot shows the proportion of 22 types of immune cells in endometriosis samples. The column of the graph is sample. **(B)** Correlation heat map of 22 immune cell infiltrates; Blue represents positive, red represents negative. The depth of the color indicates the strength of the correlation. **(C)** Changes in enrichment of 10 immune-related pathways. **(D)** Expression differences of HLA and KIR family genes.

To evaluate the extent of immune infiltration in patients with endometriosis, we used the CIBERSORT algorithm to analyze the proportions of the immune subset in endometriosis. We constructed immune cell maps (**Figure 2A**) and correlation maps of these 22 types of immune cells (**Figure 2B**) in endometriosis samples. The 22 types of immune cells are listed in **Supplementary Table 1**. We used GSVA to calculate the enrichment scores for all genes in each immune cell type. We observed changes in specific gene sets associated with 10 immune-related pathways in the c2.cp.v7.4.symbols.gmt dataset, including Toll-like receptor signaling pathway, Nod-like receptor signaling pathway, Rigi-like receptor signaling pathway, Jak-stat signaling pathway, NK cell-mediated cytotoxicity, T cell receptor signaling pathway, B cell receptor signaling pathway, autoimmune thyroid disease, allograft rejection, and graft versus host disease the gene sets and (**Figure 2C**). The HLA and KIR family genes were immune-related gene families, and the expression of each gene in the family showed different manifestations. HLA-A, HLA-B, and HLA-C showed relatively high expression, while the expression of the KIR family was relatively low (**Figure 2D**).

### 3.2 Identification of two endometriosis subtypes and exploration of DEGs based on immune characteristics

**Figure 3:**
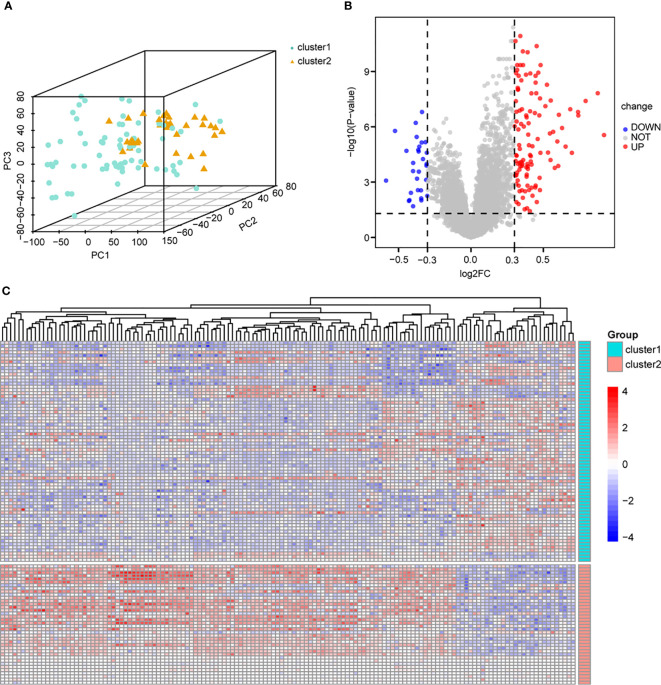
Differential analysis based on immune cell subtypes. **(A)** PCA analysis based on immune cell subtypes, cluster1 is green, cluster 2 is yellow. **(B)** Volcano map of differential analysis based on immune cell subtype, blue dots represent low expression and red dots represent high expression. **(C)** Heat map based on differential analysis of immune cell subsets, cluster1 is green, cluster 2 is red; blue dots represent low expression and red dots represent high expression. PCA, principal component analysis.

To determine the biological differences between different immune subtypes in endometriosis, we classified the endometriosis samples into cluster 1 and cluster 2 based on the consensus clustering of immune cell infiltration data. The two subtypes were clearly distinguished (**Figure 3A**). Differential gene expression was identified with limma (P-value < 0.05 and |log2FC| > 0.3). A total of 140 DEGs were obtained, including 29 low-expression genes and 111 high-expression genes (**Supplementary Table 7**). The volcano plot indicated a higher number of genes with upregulated expression compared to genes with downregulated expression (**Figure 3B**). In the heat map, the genes in cluster 1 exhibited an overall downregulated trend, while the genes in cluster 2 showed an overall upregulated trend (**Figure 3C**). GSEA and GSVA were applied for all the DEGs between cluster 1 and cluster 2, and the results were shown in **Supplementary Tables 2**, **3**, respectively. The results of GSEA were visualized in **Supplementary Figure 1A**, indicating that immunoregulatory interactions and the complement system play crucial roles in the pathology of endometriosis. The results of GSVA (**Supplementary Figure 1C**) revealed that the B cell receptor signaling pathway and CD8 TCR downstream pathway are important pathways involved in endometriosis. These findings indicate that the immunology system plays an essential role in the pathologies of endometriosis.

### 3.3 Co-expression network construction and central genes identification

**Figure 4:**
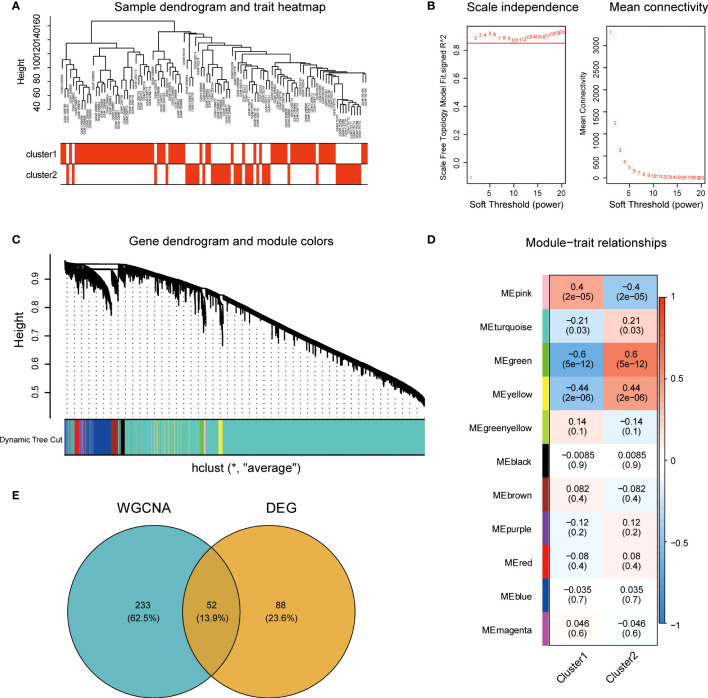
Construction and module analysis of the weighted co-expression network. **(A)** Sample clustering tree based on Euclidean distance. **(B)** Network topology analysis under various soft threshold power settings. Left: The X-axis represents the soft threshold power, and the Y-axis represents the fitting index of scale-free topological model. Right: The X-axis represents the soft threshold power, and the Y-axis reflects the average connectivity (degree). **(C)** Clustering tree of genes with different similarities based on topological overlap, and assigned module colors. **(D)** Association with Module-trait. Each row corresponds to a module, and each column corresponds to a feature. Each cell contains the corresponding correlation and P values. This table is color-coded according to the correlation of the color legends. **(E)** The intersection Venn diagram of core modules and central genes of WGCNA. WGCNA, weighted gene co-expression network based on immune cell subsets.

To explore the central genes involved in immune infiltration in endometriosis, we constructed a gene co-expression network using the WGCNA algorithm. The gene expression profile of the endometriosis immune subtype was evaluated using cluster analysis (**Figure 4A**). To ensure that the network was scale-free, we selected a soft threshold of β = 2 (**Figure 4B**). We then transformed the representation matrix into an adjacency matrix and a topological matrix and used the average linkage hierarchical clustering method to cluster the genes. We set a minimum number of 50 genes in each gene network module, based on the standard of the hybrid dynamic clipping tree. We used the dynamic shearing method to determine the gene module and calculated the characteristic gene values for each module.

We then conducted Cluster analysis on the modules and merged modules that were close to each other using the following parameters: height = 0.25, deep split = 4, and minimum module = 50, resulting in a total of 11 modules (**Figure 4C**). The module with the highest correlation with immune characteristics was the green module (r = 0.6, P = 5e-12; **Figure 4D**). We identified 285 genes in the green module. We then compared these genes to the 140 DEGs based on immune characteristics and identified 52 common genes (**Figure 4E**).

### 3.4 GO and KEGG enrichment analysis of cerntral genes in endometriosis

**Figure 5:**
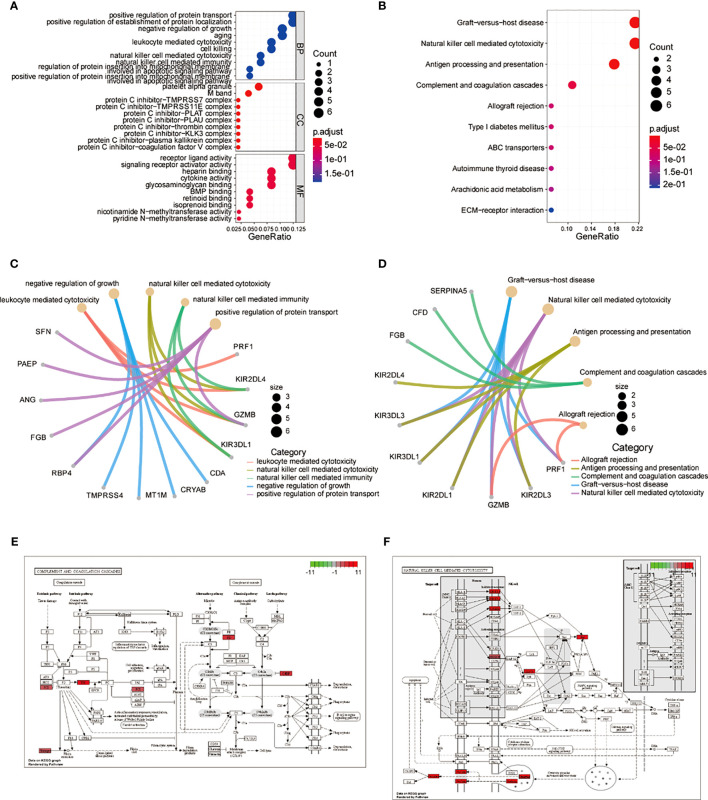
Central genes in GO, KEGG, and PATHWAY enrichment analysis. **(A)** Dot plot of central genes GO analysis. **(B)** Dot plot of central genes KEGG analysis. **(C)** Loop diagram of central genes GO analysis. **(D)** Loop diagram of central genes KEGG analysis. **(E)** Enrichment diagram of central genes in the complement and coagulation cascade pathways. **(F)** Enrichment diagram of central genes in the natural killer cell-mediated cytotoxicity pathway. GO, Gene Ontology; KEGG, Kyoto Encyclopedia of Genes and Genomes.

To further investigate the functions of the 52 common genes, we performed GO and KEGG analyses. We used a cutoff p value of 0.5 for these analyses. GO analysis showed that the common genes were mainly related to leukocyte-mediated cytotoxicity, negative regulation of growth, NK cell-mediated cytotoxicity, NK cell-mediated immunity, and positive regulation of protein transport (**Figures 5A, C**; **Supplementary Table 4**). KEGG analysis showed that the pathway was mainly related to graft-versus-host disease, NK cell-mediated cytotoxicity, antigen processing and presentation, complement and coagulation cascades, allograft rejection, and NK cells in both resting and activated states (**Figures 5B, D**; **Supplementary Table 5**). Pathway enrichment analysis also showed that the common genes were significantly expressed in the complement and coagulation cascade (**Figure 5E**) and NK cell-mediated cytotoxicity (**Figure 5F**) pathways.

### 3.5 Pathway enrichment of central genes in endometriosis

**Figure 6:**
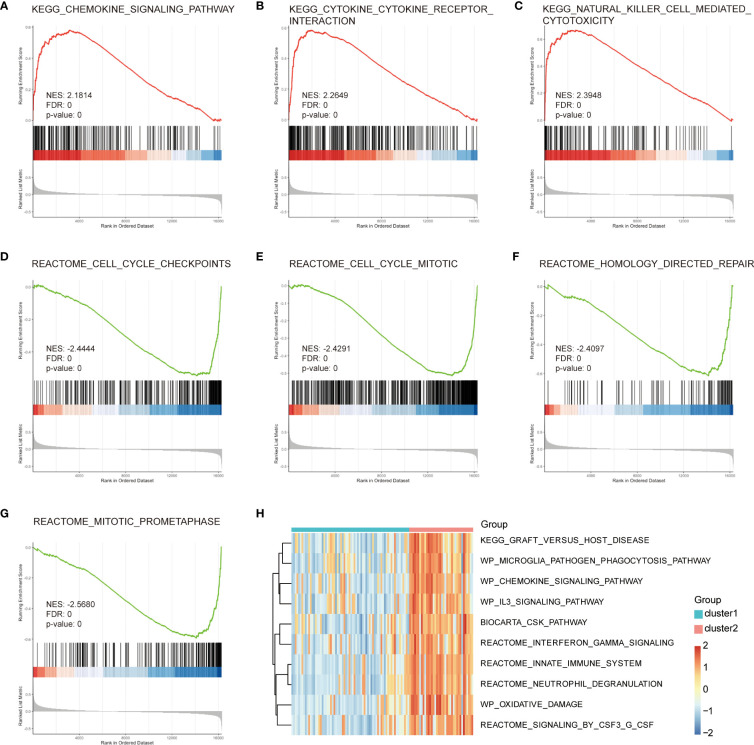
GSEA and GSVA enrichment analysis of central genes. **(A)** Cytokine–cytokine receptor interaction. **(B)** Natural killer cell-mediated cytotoxicity. **(C)** Cell cycle checkpoints. **(D)** Cell cycle mitosis. **(E)** Homology-directed repair. **(F)** Mitotic prometaphase. **(G, H)** GSVA analysis, green represents Cluster1, red represents Cluster2, small blue squares represent low expression, and small red squares represent high expression. GSEA, gene set enrichment analysis; GSVA, gene set variation analysis; BP, Biological Process; CC, Cellular Component; MF, Molecular Function.

To address potential biases in the enrichment analysis of intersection genes, we performed GSEA on all 140 DEGs related to immune subtypes in the endometriosis expression profile. We used the C2.cp.v7.4.Symbols.gmt reference gene set for this analysis. The results showed that DEGs were significantly enriched in the chemokine signaling pathway, cytokine cytokine receptor interaction, NK cell mediated cytotoxicity, cell cycle checkpoints, cell cycle mitotic, homology directed repair, and reactome mitotic prometaphase (**Figures 6A–G**). We also performed GSVA using the C2.cp.v7.4.symbols.gmt reference gene set to explore the potential mechanism underlying the pathogenesis of endometriosis. We observed significant differences in 10 gene sets based on immune subtypes, including innate immune system, biocarta csk pathway, graft versus host disease, WP oxidative damage, neutrophil degranulation, signaling by CSF3 G CSF, WP microglia pathogen phagocytosis pathway, WP chemokine signaling pathway, WP IL3 signaling pathway, and interferon-gamma signaling. Most of these DEG sets were immune-related pathways (**Figure 6H**).

### 3.6 Construction of PPI network

To explore the protein functions of the 52 candidate genes (**Supplementary Table 6**), we constructed a PPI network using the STRING database and Cytoscape software. With a confidence score of 0.55, we identified 45 genes that showed close interactions with each other (**Supplementary Figure 2A**), while only 8 genes remained with a cut-off of > 0.7 (**Supplementary Figure 3**). We utilized an interaction score of 0.55 to identify the hub genes. Using the cytoHubba plugin, we calculated the value of the PPI network and selected the top 10 hub genes (**Supplementary Figure 2B**) and their extensions: GZMB, PRF1, KIR2DL1, KIR2DL3, KIR3DL1, KIR2DL4, FGB, IGFBP1, RBP4, and PROK1 (**Supplementary Figure 2C**). To further explore the upstream regulatory relationships, we predicted miRNA interactions with the hub gene by selecting the results of “Functional MTI” and “positive” evidence, based on the multiMiR and TarBase v.8 databases. We found that 8 mRNAs in the hub genes interacted with 11 miRNAs (**Supplementary Figure 2D**).

### 3.7 Identification of three endometriosis subtypes through unsupervised clustering based on hub genes

**Figure 7:**
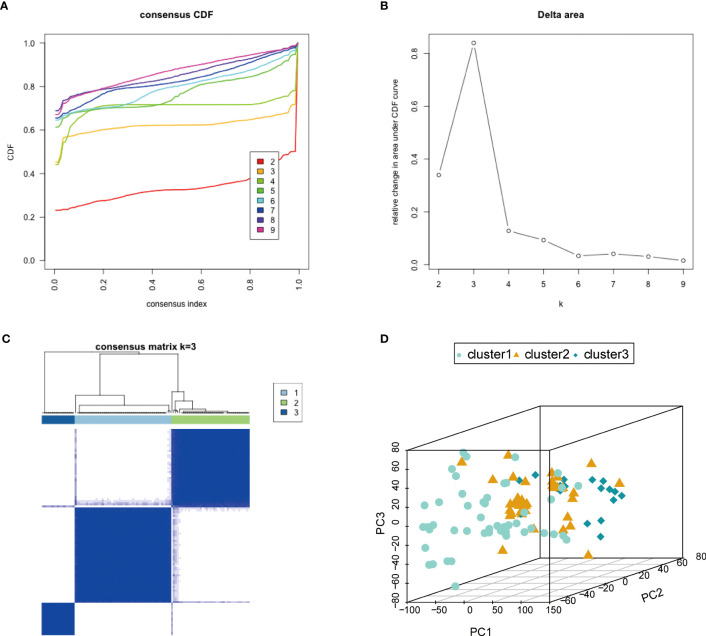
Characteristics based on endometriosis-related molecular typing. **(A)** Consensus CDF. **(B)** Delta region. **(C)** Consistent matrix at k = 3. The rows and columns of the matrix represent samples. **(D)** The PCA diagram verifies the stability and reliability of classification. PCA, principal component analysis.

To investigate the biological differences among different subtypes based on the characteristics of endometriosis, we used the ConsensusClusterPlus software package to construct subtypes based on the expression profile of the 10 hub genes. When k = 3, the classification was reliable and stable (**Figures 7A–C**). The samples were divided into cluster 1, cluster 2, and cluster 3. PCA confirmed that cluster 1, cluster 2, and cluster 3 showed significant differences (**Figure 7D**). We also analyzed the correlation of immune cell fractions by disease subtypes and found that the three components showed different expressions in different immune cells. NK.cells.activated, T.cells.CD8, and NK.cells.resting showed a large difference in expression (**Supplementary Figure 4G**). For the hub genes, we calculated the correlation between the hub gene expression and immune cell fraction (Pearson’s coefficient). Most hub genes were significantly correlated with immune markers. We observed a significant positive correlation between GZMB and NK.cells.activated (P = 1.23e-11, R = 0.59) (**Supplementary Figure 4A**), KIR2DL1 and NK.cells.resting (P = 2.51e-8, R = 0.50) (**Supplementary Figure 4B**), KIR2DL3 and NK.cells.resting (P = 1.55e-11, R = 0.59) (**Supplementary Figure 4C**), KIR2DL4 and NK.cells.resting (P = 4.26e-12, R = 0.60) (**Supplementary Figure 4D**), PRF1 and NK.cells.activated (P = 3.54e-10, R = 0.56) (**Supplementary Figure 4E**), and PRF1 and NK.cells.resting (P = 1.15e-11, R = 0.59) (**Supplementary Figure 4F**). Further analysis of the correlation between hub genes and immune checkpoint genes revealed that hub genes were strongly positively correlated with CD40, IDO1, LAG3, TNF, and TNFRSF18 immune checkpoint genes (**Supplementary Figure 4H**). These results showed that the expression of hub genes was significantly positively correlated with immune characteristics.

### 3.8 Expression validation of hub genes

**Figure 8:**
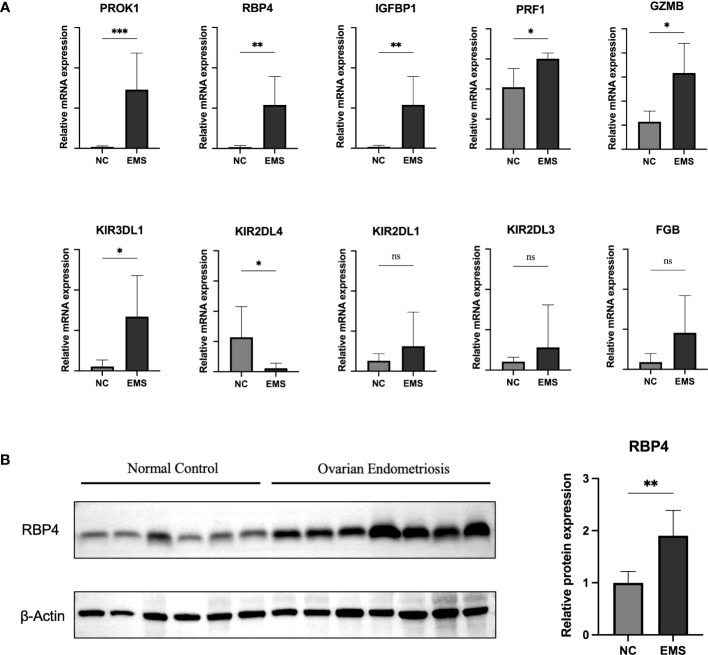
Expression validation of hub genes. **(A)** qRT-PCR was performed to determine mRNA expression of hub genes in normal eutopic endometrial tissue and ovarian endometriosis tissue. **(B)** Western blotting was performed to determine the protein expression of RBP4 in normal eutopic endometrial tissue and ovarian endometriosis tissue. EMS, endometriosis. Data are expressed as the mean ± SEM. *P < 0.05, **P < 0.01; ns, non-significant.

To validate the transcriptional and protein expression of the hub genes, we used qRT‐PCR and Western blot analyses to detect their expression in both ovarian endometriosis tissue and normal eutopic endometrial tissue from the same patients (**Figure 8**). We found that, except for FGB, KIR2DL1, and KIR2DL3, all other hub genes showed significantly different expression levels in the endometriosis and normal control groups (**Figure 8A**). Specifically, the expression of KIR2DL4 was remarkably downregulated, while the expressions of PROK1, IGFBP1, RBP4, G2MB, KIR3DL1, and PRF1 were significantly upregulated in ovarian endometriosis. Moreover, the protein expression of RBP4 was remarkably higher in ovarian endometriosis than in normal control (**Figure 8B**).

## 4 Discussion

Endometriosis is a common disease that affects up to 10% of women. It is characterized by pain and infertility (31). Up to 90% of cases of chronic pelvic pain in women of reproductive age are associated with endometriosis (32). Endometriosis is also a risk factor for ovarian cancer (33). However, endometriosis is not curable, and its underlying etiology remains unclear. In a previous study, we identified PDLIM3 as a specific biomarker for endometriosis that was associated with multiple immune cells (15). However, that analysis focused on the relationship between a single gene and the immune cell environment.

In the present study, we conducted a more comprehensive analysis of the immune cell environment and explored immune-associated genes in endometriosis. We merged three mRNA microarray datasets (GSE51981, GSE6364, GSE7305) into one dataset with 108 samples of endometriosis and conducted several analyses. Our findings indicated that hub genes, enriched modules, and pathways have genetic effects on endometriosis. Moreover, the predicted genes in the merged dataset might interact with each other and coregulate endometriosis.

HLA and KIR are immune-related gene families. KIRs are expressed by NK cells and can recognize class I HLA on target cells. HLA-C is a ligand of KIRs and is an essential regulator of NK cell activity (34, 35). Modulation of the HLA-KIR axis provides new prospects for cancer treatment (36) and immunotherapy of other diseases such as COPD (37). A previous study revealed that the expression of HLA-C*03:03*01 was increased in endometriosis. Aberrant expression of KIR2DL1 impaired NK cell cytotoxicity in endometriosis (38). KIR2DS5-positive women with endometriosis had a lesser chance of peritoneal disease, and KIR2DS5 might be a protective factor for endometriosis (34). In this study, we constructed 22 immune profiles from endometriosis samples. GSVA was used to calculate enrichment scores for specific gene sets in each sample, and variations of 10 immune pathways, including Toll-like receptor signaling pathway, Nod-like receptor signaling pathway, and Rig I-like receptor signaling pathway, were observed. HLA-A, HLA-B, and HLA-C expression levels were relatively high, and KIR expression was relatively low, which is consistent with previous reports (34, 35).

To investigate the biological functions of the differentially expressed genes, we analyzed 52 common genes that were common to both immunogrouping and WGCNA analyses and found several functions were associated with endometriosis, including leukocyte-mediated cytotoxicity, negative regulation of growth, NK cell-mediated cytotoxicity, NK cell-mediated immunity, and positive regulation of protein transport. Further analysis using GO and KEGG pathways showed that the complement and coagulation cascade pathways and NK cell-mediated cytotoxicity pathways were significantly associated with endometriosis. Consistent with our findings, the abnormal immune function of NK cells is closely related to endometriosis (39). In particular, impaired NK cell cytotoxic activity has been linked to the development of endometriosis (40, 41). To validate our results, we performed GSEA and GSVA analysis on differential gene sets related to immune subtypes. Our analysis showed common enrichment in NK cell-mediated cytotoxicity and graft versus host disease, and most differential gene sets were immune-related pathways. These results further support the important role of NK cells in the development of endometriosis.

The immune system plays a crucial role in the pathogenesis of endometriosis, with immune cells infiltrating the ectopic endometrial lesions. Immune infiltration points in endometriosis include the involvement of various immune cell types, such as macrophages, neutrophils, NK cells, and CD8^+^ T cells. Macrophages, found in increased numbers in the peritoneal fluid and at the site of the lesions, contribute to the development and maintenance of endometriotic lesions by producing growth factors, angiogenic factors, and pro-inflammatory cytokines (42–44). Previous study suggests that M2 macrophages may play a role in the development and recurrence of endometriosis (45). Additionally, microvesicles secreted by M1 macrophages in endometriosis can induce polarization of M2 macrophages towards an M1 phenotype, potentially inhibiting the development of endometriosis (46). These studies indicate the presence of both M1 and M2 macrophages in endometriotic lesions and suggest that repolarizing M2 macrophages towards an M1 phenotype could be a potential therapeutic strategy. However, further research and clinical studies are needed to fully understand the role of macrophage subtypes and their potential for the treatment of endometriosis. Similarly, neutrophils are present in the peritoneal fluid of women with endometriosis and promote angiogenesis and tissue remodeling in the formation of endometriotic lesions (47, 48). Moreover, NK cells in peripheral blood might help to destroy retrograde endometrial cells, thus preventing endometriosis development. The reduction in NK cell cytotoxic activity might result in endometriosis (49). The alterations in the immunological parameters of NK cells not only included lower NK cell cytotoxicity but also involved a shift in the balance between type 1 and type 2 NK cells, as well as changes in the percentages of inhibitory and activating NK cell receptors (39). Thus, abnormalities in the function of NK cells, such as reduced cytotoxicity, altered activity and phenotype, and imbalances in receptor expression, might contribute to the development and pathology of endometriosis. In our study, the NK cell-mediated cytotoxicity pathway is implicated in several analyses, aligning with previous findings. CD8 T cells also play a role in the development and progression of endometriosis. An increased number of CD8 T cells has been observed in the peritoneal fluid of women with endometriosis compared to those without the disease, suggesting a possible role of CD8 T cells in the local immune response (50). CD8 T cells in the peritoneal fluid of endometriosis patients exhibited a reduced cytotoxic capacity, potentially contributing to impaired immune surveillance and facilitating the establishment of endometrial implants in the peritoneal cavity (51).

To further elucidate the pathogenesis of endometriosis, we performed PPI analyses to identify the endometriosis-associated hub genes. We selected 10 DEGs as hub genes, namely, GZMB, PRF1, KIR2DL1, KIR2DL3, KIR3DL1, KIR2DL4, FGB, IGFBP1, RBP4, and PROK1. Among these, KIR2DL1, KIR2DL3, KIR3DL1, and KIR2DL4 belong to NK cell inhibitory receptors (39). Their functions are mentioned above. GZMB is a component of cytolytic granules within NK cells (52) and was reported to be correlated with cervical cancer (53). RBP4 belongs to the lipocalin family and is the major transport protein of the hydrophobic molecule retinol, which is known as vitamin A (54). A recent study showed that RBP4 is involved in the pathological process of endometriosis and could promote the activity, proliferation, and invasion of ectopic cells (55). We validated that RBP4 was overexpressed in endometriosis patients. In addition, there are no reports about RBP4 and other uterine pathologies, which indicates that this signature is unique to endometriosis. PROK1 is a secreted peptide that belongs to the prokineticin family and performs a wide range of functions in angiogenesis, modulation of inflammatory responses, and regulation of hematopoiesis (56). Tiberi et al. discovered that the expression of PROK1 in normal female tissues was much higher than that in women with endometriosis, suggesting that women with endometriosis might show abnormal vascular function (57).

In our study, all the hub genes were found to be highly correlated with endometriosis. For example, GZMB (Granzyme B) belongs to the granzyme family of serine proteases that are found in the cytotoxic granules of cytotoxic T lymphocytes and NK cells (58). Granzymes play a critical role in the immune system by inducing apoptosis in target cells, such as virally infected cells or tumor cells (59). GZMB is a therapeutic target for endometriosis, based on its role in immune response, tissue remodeling, and angiogenesis. Targeting GZMB might alleviate endometriosis-associated pain and inflammation while preserving fertility (60). Apart from their association with endometriosis, some of the hub genes have also been linked to other uterine pathologies. For instance, the KIR family (61), IGFBP1 (62), and PROK1 (63) have been correlated with recurrent implantation failure, while GZMB (64) and FGB (65) have been implicated in cervical carcinoma. However, there is no report about the relationship between PRF1 and RBP4 and other uterine pathologies, which suggests that these two might be unique signatures of endometriosis.

To further explore upstream regulatory relationships, multiMiR database, and TarBase V. 8 database were used to predict miRNAs interacting with hub genes. Eight mRNAs in hub genes were found to interact with 11 miRNAs, namely, miR-29c-3p, miR-542-3p, miR-27a-3p, miR-31-5p, miR-16-5p, miR-29b-3p, miR-124-3p, miR-21-5p, miR-409-3p, miR-107, and miR-452-5p. MiRNAs could inhibit the translation of their target genes by binding to their messenger RNA 3′-untranslated region (66). This posttranscriptional regulation occurs during physiopathologic processes, including endometriosis (67). Changes in the function of mir-542-3p and its target gene IGFBP1 have been reported to alter the decidualization of endometriosis stromal cells, affecting the metastasis and invasion of ectopic endometriosis cells (68, 69). Braza-Boïls et al. indicated that many anomalously expressed miRNAs were identified in peritoneal fluid, including miR-16-5p. These miRNAs affected the expression of VEGF-A and had an important influence on the occurrence and development of endometriosis (70). Overall, these results are similar to those from our mining data.

Our study has some limitations. Firstly, as the pathogenesis of endometriosis is multidimensional, discussing it only from the aspect of immune infiltration might not be comprehensive enough. Secondly, all study data were obtained from publicly available databases, and additional clinical characteristics of endometriosis patients should be included in subgroup analysis. Thirdly, although we validated the expression of hub genes through qRT-PCR and western blot, further multicenter and prospective studies are required to evaluate the possible applications of molecular signatures. Additionally, more *in vivo* and *in vitro* experiments are needed to elucidate the molecular mechanisms of hub genes for clinical applications. Furthermore, while we have analyzed gene expression data for HLA and KIR genes, we acknowledge that further investigation in specific cell types and disease contexts through sequencing or genotyping approaches is necessary to better understand the heterogeneity of these gene families. Lastly, scRNA-seq analysis may provide more valuable insights into immune cell heterogeneity compared to bulk RNA-seq, and we plan to conduct scRNA-seq analysis in future studies.

This study presents several novel aspects compared to previous similar research. Firstly, we incorporate a novel approach by considering the immune phenotype of patients with endometriosis. By analyzing the immune cell composition and immune-related pathways in endometriosis, we provide valuable insights into immune dysregulation associated with the disease. Secondly, we utilize diverse datasets to comprehensively analyze the immune landscape, revealing new relationships between immune cell subgroups, gene expression patterns, and immune pathways in endometriosis. Advanced analysis methods, such as CIBERSORT, GSVA, and WGCNA, further deepened the exploration, identifying key genes associated with immune subtypes. Notably, the study uncovers two previously unknown genes, KIR2DL3 and FGB, in endometriosis, contributing to the understanding of potential genetic factors in the disease.

In conclusion, this study provides comprehensive and reliable evidence for the pathogenesis of endometriosis from the perspective of immune infiltration. The results showed that most of the DEGs are involved in immune-related pathways. Ten hub genes, which show significant correlation with immune markers, might be potential diagnostic and therapeutic targets for endometriosis.

Our study provided a reliable and deep analysis of the mechanism underlying endometriosis from the perspective of immune infiltration. A total of 10 hub genes were identified and were validated by qRT-PCR and western blotting. The majority of hub genes showed significantly different expression between endometriosis patients and normal controls. Of these genes, PROK1 (Prokineticin 1) has been suggested to be associated with endometriosis due to its role in promoting angiogenesis (71). Altered expression of IGFBP1 (Insulin-like growth factor-binding protein 1) has been found in the endometrium of women with endometriosis, suggesting a potential association (72). KIR2DL4 and KIR3DL1 are members of the killer cell immunoglobulin-like receptor family and might be associated with the pathogenesis of endometriosis through their involvement in the regulation of NK cell activity (35). No direct link has been established between RBP4, G2MB, or PRF1 and endometriosis, necessitating further investigation.

## Data availability statement

The original contributions presented in the study are included in the article/**Supplementary Material**. Further inquiries can be directed to the corresponding author.

## Ethics statement

The studies involving humans were approved by Ethics Committee of Shanghai First Maternity and Infant Hospital. The studies were conducted in accordance with the local legislation and institutional requirements. The participants provided their written informed consent to participate in this study.

## Author contributions

JS: Conceptualization, Supervision. S-JL: Project administration, Formal analysis, Investigation, Methodology, Writing -original draft J-NS: Software, Investigation, Visualization, Writing -review & editing, Validation. LG: Data curation, Resources. All authors contributed to the article and approved the submitted version.

## Supplementary material

Supplementary Figure 1 Pathways enrichment of DEGs in endometriosis. **(A)** GSEA results in the endometriosis group. **(B)** GSEA results in the control group. **(C)** Heatmap of the enriched pathways. DEGs, differentially expressed genes; GSEA, gene set enrichment analysis.

Supplementary Figure 2 PPI network and miRNA-mRNA network analysis. **(A)** PPI network of 45 common genes constructed by the STRING database. The higher the degree value of the genes, the darker the color and the larger the diameter. **(B)** Top 10 hub genes and their extensions calculated by cytoHubba. Extension genes were green. The higher enrichment scores were indicated by darker colors. **(C)** The top 10 hub genes calculated by cytoHubba. The higher enrichment scores were indicated by darker colors. **(D)** miRNA-mRNA interaction network. Green circles represent mRNA and brown triangles represent miRNAs. PPI, Protein-Protein Interaction.

Supplementary Figure 3 PPI network with an interaction score of 0.7. PPI: Protein-Protein Interaction.

Supplementary Figure 4 Correlation analysis of key genes, immune cell infiltration level, and molecular subtypes. **(A)** Correlation point plot between GZMB and NK.cells.activated (P = 1.23e-11, R = 0.59). **(B)** Correlation point plot between KIR2DL1 and NK.cells.resting (P = 2.51e-8, R = 0.50). **(C)** Correlation point plot between KIR2DL3 and NK.cells.resting (P = 1.55e-11, R = 0.59). **(D)** Correlation point plot between KIR2DL4 and NK.cells.resting (P = 4.26e-12, R = 0.60). **(E)** Correlation point plot between PRF1 and NK.cells.activated (P = 3.54e-10, R = 0.56). **(F)** Correlation point plot between PRF1 and NK.cells.resting (P = 1.15e-11, R = 0.59). **(G)** Correlation analysis of 22 immune cell fractions in 3 disease subtypes. **(H)** Correlation between hub genes and immune checkpoint genes.

Supplementary Table 1 The list of 22 immune cells.

Supplementary Table 2 The GSEA analysis results.

Supplementary Table 3 The GSVA analysis results.

Supplementary Table 4 GO analysis of central genes.

Supplementary Table 5 KEGG analysis of central genes.

Supplementary Table 6 The list of candidate targets for STRING analysis.

## References

[1] Noh EJ Kim DJ Lee JY Park JH Kim JS Han JW. Ureaplasma urealyticum infection contributes to the development of pelvic endometriosis through toll-like receptor 2. Front Immunol (2019) 10:2373. doi: 10.3389/fimmu.2019.02373 31636643 PMC6788432

[2] Zondervan KT Becker CM Missmer SA. Endometriosis. N Engl J Med (2020) 382(13):1244–56. doi: 10.1056/NEJMra1810764 32212520

[3] Macer ML Taylor HS. Endometriosis and infertility: a review of the pathogenesis and treatment of endometriosis-associated infertility. Obstet Gynecol Clin North Am (2012) 39(4):535–49. doi: 10.1016/j.ogc.2012.10.002 PMC3538128 23182559

[4] Zondervan KT Becker CM Koga K Missmer SA Taylor RN Vigano P. Endometriosis. Nat Rev Dis Primers (2018) 4(1):9. doi: 10.1038/s41572-018-0008-5 30026507

[5] Lin X Dai Y Tong X Xu W Huang Q Jin X. Excessive oxidative stress in cumulus granulosa cells induced cell senescence contributes to endometriosis-associated infertility. Redox Biol (2020) 30:101431. doi: 10.1016/j.redox.2020.101431 31972508 PMC6974790

[6] Taylor HS Kotlyar AM Flores VA. Endometriosis is a chronic systemic disease: clinical challenges and novel innovations. Lancet (2021) 397(10276):839–52. doi: 10.1016/S0140-6736(21)00389-5 33640070

[7] Kiesel L Sourouni M. Diagnosis of endometriosis in the 21st century. Climacteric (2019) 22(3):296–302. doi: 10.1080/13697137.2019.1578743 30905186

[8] Falcone T Flyckt R. Clinical management of endometriosis. Obstet Gynecol (2018) 131(3):557–71. doi: 10.1097/AOG.0000000000002469 29420391

[9] Ianieri MM Mautone D Ceccaroni M. Recurrence in deep infiltrating endometriosis: A systematic review of the literature, J Minim Invasive Gynecol (2018) 25(5):786–93. doi: 10.1016/j.jmig.2017.12.025 29357317

[10] Ichimiya M Hirota T Muto M. Intralymphatic embolic cells with cutaneous endometriosis in the umbilicus. J Dermatol (1998) 25(5):333–6. doi: 10.1111/j.1346-8138.1998.tb02407.x 9640888

[11] Othman Eel D Hornung D Salem HT Khalifa EA El-Metwally TH Al-Hendy A. Serum cytokines as biomarkers for nonsurgical prediction of endometriosis. Eur J Obstet Gynecol Reprod Biol (2008) 137(2):240–6. doi: 10.1016/j.ejogrb.2007.05.001 17582674

[12] Shigesi N Kvaskoff M Kirtley S Feng Q Fang H Knight JC. The association between endometriosis and autoimmune diseases: a systematic review and meta-analysis. Hum Reprod Update (2019) 25(4):486–503. doi: 10.1093/humupd/dmz014 31260048 PMC6601386

[13] Wu XG Chen JJ Zhou HL Wu Y Lin F Shi J. Identification and validation of the signatures of infiltrating immune cells in the eutopic endometrium endometria of women with endometriosis. Front Immunol (2021) 12:671201. doi: 10.3389/fimmu.2021.671201 34539624 PMC8446207

[14] Zhong Q Yang F Chen X Li J Zhong C Chen S. Patterns of immune infiltration in endometriosis and their relationship to r-AFS stages. Front Genet (2021) 12:631715. doi: 10.3389/fgene.2021.631715 34220927 PMC8249861

[15] Gan L Sun J Sun J. Bioinformatical analysis identifies PDLIM3 as a potential biomarker associated with immune infiltration in patients with endometriosis. PeerJ (2022) 10:e13218. doi: 10.7717/peerj.13218 35378934 PMC8976475

[16] Davis S Meltzer PS. GEOquery: a bridge between the Gene Expression Omnibus (GEO) and BioConductor. Bioinformatics (2007) 23(14):1846–7. doi: 10.1093/bioinformatics/btm254 17496320

[17] Newman AM Liu CL Green MR Gentles AJ Feng W Xu Y. Robust enumeration of cell subsets from tissue expression profiles. Nat Methods (2015) 12(5):453–7. doi: 10.1038/nmeth.3337 PMC4739640 25822800

[18] Wickham H. ggplot2 : Elegant Graphics for Data Analysis. In: Use R!. Cham: Springer International Publishing : Imprint: Springer (2016).

[19] Subramanian A Tamayo P Mootha VK Mukherjee S Ebert BL Gillette MA. Gene set enrichment analysis: a knowledge-based approach for interpreting genome-wide expression profiles. Proc Natl Acad Sci USA (2005) 102(43):15545–50. doi: 10.1073/pnas.0506580102 PMC1239896 16199517

[20] Hanzelmann S Castelo R Guinney J. GSVA: gene set variation analysis for microarray and RNA-seq data. BMC Bioinf (2013) 14:7. doi: 10.1186/1471-2105-14-7 PMC3618321 23323831

[21] Wilkerson MD Hayes DN. ConsensusClusterPlus: a class discovery tool with confidence assessments and item tracking. Bioinformatics (2010) 26(12):1572–3. doi: 10.1093/bioinformatics/btq170 PMC2881355 20427518

[22] Ritchie ME Phipson B Wu D Hu Y Law CW Shi W. limma powers differential expression analyses for RNA-sequencing and microarray studies. Nucleic Acids Res (2015) 43(7):e47. doi: 10.1093/nar/gkv007 25605792 PMC4402510

[23] Liang JW Fang ZY Huang Y Liuyang ZY Zhang XL Wang JL. Application of weighted gene co-expression network analysis to explore the key genes in alzheimer's disease. J Alzheimers Dis (2018) 65(4):1353–64. doi: 10.3233/JAD-180400 PMC6218130 30124448

[24] Yu G Wang LG Han Y He QY. clusterProfiler: an R package for comparing biological themes among gene clusters. OMICS (2012) 16(5):284–7. doi: 10.1089/omi.2011.0118 PMC3339379 22455463

[25] Luo W Brouwer C. Pathview: an R/Bioconductor package for pathway-based data integration and visualization. Bioinformatics (2013) 29(14):1830–1. doi: 10.1093/bioinformatics/btt285 PMC3702256 23740750

[26] Szklarczyk D Gable AL Lyon D Junge A Wyder S Huerta-Cepas J. STRING v11: protein-protein association networks with increased coverage, supporting functional discovery in genome-wide experimental datasets. Nucleic Acids Res (2019) 47(D1):D607–13. doi: 10.1093/nar/gky1131 PMC6323986 30476243

[27] Shannon P Markiel A Ozier O Baliga NS Wang JT Ramage D. Cytoscape: a software environment for integrated models of biomolecular interaction networks. Genome Res (2003) 13(11):2498–504. doi: 10.1101/gr.1239303 PMC403769 14597658

[28] Chin CH Chen SH Wu HH Ho CW Ko MT Lin CY. cytoHubba: identifying hub objects and sub-networks from complex interactome. BMC Syst Biol (2014) 8 Suppl 4:S11. doi: 10.1186/1752-0509-8-S4-S11 25521941 PMC4290687

[29] Friendly M. Corrgrams: Exploratory displays for correlation matrices. Am Statistician (2002) 56(4):316–24. doi: 10.1198/000313002533

[30] Ru Y Kechris KJ Tabakoff B Hoffman P Radcliffe RA Bowler R. The multiMiR R package and database: integration of microRNA-target interactions along with their disease and drug associations. Nucleic Acids Res (2014) 42(17):e133. doi: 10.1093/nar/gku631 25063298 PMC4176155

[31] Lalami I Abo C Borghese B Chapron C Vaiman D. Genomics of endometriosis: from genome wide association studies to exome sequencing. Int J Mol Sci (2021) 22(14):7297. doi: 10.3390/ijms22147297 34298916 PMC8304276

[32] McAllister SL Sinharoy P Vasu M Gross ER. Aberrant reactive aldehyde detoxification by aldehyde dehydrogenase-2 influences endometriosis development and pain-associated behaviors. Pain (2021) 162(1):71–83. doi: 10.1097/j.pain.0000000000001949 32541390 PMC7718385

[33] Lin SC Lee HC Hsu CT Huang YH Li WN Hsu PL. Targeting anthrax toxin receptor 2 ameliorates endometriosis progression. Theranostics (2019) 9(3):620–32. doi: 10.7150/thno.30655 PMC6376465 30809297

[34] Nowak I Ploski R Barcz E Dziunycz P Kaminski P Kostrzewa G. KIR2DS5 in the presence of HLA-C C2 protects against endometriosis. Immunogenetics (2015) 67(4):203–9. doi: 10.1007/s00251-015-0828-3 PMC4357646 25724317

[35] Chou YC Chen CH Chen MJ Chang CW Chen PH Yu MH. Killer cell immunoglobulin-like receptors (KIR) and human leukocyte antigen-C (HLA-C) allorecognition patterns in women with endometriosis. Sci Rep (2020) 10(1):4897. doi: 10.1038/s41598-020-61702-y 32184413 PMC7078270

[36] Pende D Falco M Vitale M Cantoni C Vitale C Munari E. Killer ig-like receptors (KIRs): Their role in NK cell modulation and developments leading to their clinical exploitation. Front Immunol (2019) 10:1179. doi: 10.3389/fimmu.2019.01179 31231370 PMC6558367

[37] Mkorombindo T Tran-Nguyen T Yuan K Zhang Y Xue J Criner GJ. HLA-C and KIR permutations influence chronic obstructive pulmonary disease risk. JCI Insight (2021) 6(19):e150187. doi: 10.1172/jci.insight.150187 34464355 PMC8525585

[38] Du Y Liu X Guo SW. Platelets impair natural killer cell reactivity and function in endometriosis through multiple mechanisms. Hum Reprod (2017) 32(4):794–810. doi: 10.1093/humrep/dex014 28184445

[39] Fukui A Mai C Saeki S Yamamoto M Takeyama R Kato T. Pelvic endometriosis and natural killer cell immunity. Am J Reprod Immunol (2021) 85(4):e13342. doi: 10.1111/aji.13342 32896016

[40] Maeda N Izumiya C Yamamoto Y Oguri H Kusume T Fukaya T. Increased killer inhibitory receptor KIR2DL1 expression among natural killer cells in women with pelvic endometriosis. Fertil Steril (2002) 77(2):297–302. doi: 10.1016/s0015-0282(01)02964-8 11821086

[41] Mei J Zhou WJ Zhu XY Lu H Wu K Yang HL. Suppression of autophagy and HCK signaling promotes PTGS2(high) FCGR3(-) NK cell differentiation triggered by ectopic endometrial stromal cells. Autophagy (2018) 14(8):1376–97. doi: 10.1080/15548627.2018.1476809 PMC6103661 29962266

[42] Bacci M Capobianco A Monno A Cottone L Di Puppo F Camisa B. Macrophages are alternatively activated in patients with endometriosis and required for growth and vascularization of lesions in a mouse model of disease. Am J Pathol (2009) 175(2):547–56. doi: 10.2353/ajpath.2009.081011 PMC2716955 19574425

[43] Capobianco A Rovere-Querini P. Endometriosis, a disease of the macrophage. Front Immunol (2013) 28:9. doi: 10.3389/fimmu.2013.00009 PMC3556586 23372570

[44] Chen S Liu Y Zhong Z Wei C Liu Y Zhu X. Peritoneal immune microenvironment of endometriosis: Role and therapeutic perspectives. Front Immunol (2023) 14:1134663. doi: 10.3389/fimmu.2023.1134663 36865552 PMC9971222

[45] Sun SG Guo JJ Qu XY Tang XY Lin YY Hua KQ. The extracellular vesicular pseudogene LGMNP1 induces M2-like macrophage polarization by upregulating LGMN and serves as a novel promising predictive biomarker for ovarian endometriosis recurrence. Hum Reprod (2022) 37(3):447–65. doi: 10.1093/humrep/deab266 34893848

[46] Li QJ Yuan M Jiao X Huang YF Li J Li D. M1 macrophage-derived nanovesicles repolarize M2 macrophages for inhibiting the development of endometriosis. Front Immunol (2021) 12:707784. doi: 10.3389/fimmu.2021.707784 34354711 PMC8329654

[47] Gmyrek GB Sozanski R Jerzak M Chrobak A Wickiewicz D Skupnik A. Evaluation of monocyte chemotactic protein-1 levels in peripheral blood of infertile women with endometriosis. Eur J Obstet Gynecol Reprod Biol (2005) 122(2):199–205. doi: 10.1016/j.ejogrb.2005.03.019 15893866

[48] Santulli P Lamau MC Marcellin L Gayet V Marzouk P Borghese B. Endometriosis-related infertility: Ovarian endometrioma per se is not associated with presentation for infertility. Hum Reprod (2016) 8:1765–75. doi: 10.1093/humrep/dew093 27130614

[49] Osuga Y Koga K Hirota Y Hirata T Yoshino O Taketani Y. Lymphocytes in endometriosis. Am J Reprod Immunol (2011) 65(1):1–10. doi: 10.1111/j.1600-0897.2010.00887.x 20584009

[50] Berkkanoglu M Arici A. Immunology and endometriosis. Am J Reprod Immunol (2003) 50(1):48–59. doi: 10.1034/j.1600-0897 14506928

[51] Podgaec S Abrao MS Dias JAi Jr. Rizzo LV de Oliveira RM Baracat EC. Endometriosis: An inflammatory disease with a th2 immune response component. Hum Reprod (2007) 22(5):1373–9. doi: 10.1093/humrep/del516 17234676

[52] Oboshi W Watanabe T Hayashi K Nakamura T Yukimasa N. QPY/RAH haplotypes of the GZMB gene are associated with natural killer cell cytotoxicity. Immunogenetics (2018) 70(1):29–36. doi: 10.1007/s00251-017-1014-6 28653095

[53] Zhou C Li C Zheng Y Liu X. Identification of pyroptosis-related signature for cervical cancer predicting prognosis. Aging (Albany NY) (2021) 13(22):24795–814. doi: 10.18632/aging.203716 PMC8660613 34837692

[54] Steinhoff JS Lass A Schupp M. Biological functions of RBP4 and its relevance for human diseases. Front Physiol (2021) 12:659977. doi: 10.3389/fphys.2021.659977 33790810 PMC8006376

[55] Lee JC Kim SH Oh YS Kim JH Lee SR Chae HD. Increased expression of retinol-binding protein 4 in ovarian endometrioma and its possible role in the pathogenesis of endometriosis. Int J Mol Sci (2021) 22(11):5827. doi: 10.3390/ijms22115827 34072419 PMC8199072

[56] Su MT Huang JY Tsai HL Chen YC Kuo PL. A common variant of PROK1 (V67I) acts as a genetic modifier in early human pregnancy through down-regulation of gene expression. Int J Mol Sci (2016) 17(2):162. doi: 10.3390/ijms17020162 26828479 PMC4783896

[57] Tiberi F Tropea A Apa R ROmani F Lanzone A Marana R. Prokineticin 1 mRNA expression in the endometrium of healthy women and in the eutopic endometrium of women with endometriosis. Fertil Steril (2010) 93(7):2145–9. doi: 10.1016/j.fertnstert.2009.01.105 19285664

[58] Afonina IS Tynan GA Logue SE Cullen SP Bots M Luthi AU. Granzyme B-dependent proteolysis acts as a switch to enhance the proinflammatory activity of il-1alpha. Mol Cell (2011) 44(2):265–78. doi: 10.1016/j.molcel.2011.07.037 PMC3319689 22017873

[59] Trapani JA. Granzymes: A family of lymphocyte granule serine proteases. Genome Biol (2001) 2(12):REVIEWS3014. doi: 10.1186/gb-2001-2-12-reviews3014 11790262 PMC138995

[60] Aubert A Lane M Jung K Granville DJ. Granzyme B as a therapeutic target: An update in 2022. Expert Opin Ther Targets (2022) 26(11):979–93. doi: 10.1080/14728222.2022.2161890 36542784

[61] Nowak I Wilczynska K Wilczynski JR Malinowski A Radwan P Radwan M. KIR, LILRB and their ligands' Genes as potential biomarkers in recurrent implantation failure. Arch Immunol Ther Exp (Warsz) (2017) 65(5):391–9. doi: 10.1007/s00005-017-0474-6 PMC5602049 28523429

[62] Fu YX Yang HM OuYang XE Hu R Hu T Wang FM. Assessment of anti-mullerian hormone and anti-mullerian hormone type II receptor variants in women with repeated implantation failures. Reprod Sci (2021) 28(2):406–15. doi: 10.1007/s43032-020-00303-6 32845508

[63] Karaer A Tuncay G Uysal O Semerci Sevimli T Sahin N Karabulut U. The role of prokineticins in recurrent implantation failure. J Gynecol Obstet Hum Reprod (2020) 49(9):101835. doi: 10.1016/j.jogoh.2020.101835 32585394

[64] Guzman VB Silva ID Brenna SM Carvalho CR Ribalta JC Gerbase-Delima M. High levels of granzyme B expression in invasive cervical carcinoma correlates to poor response to treatment. Cancer Invest (2008) 26(5):499–503. doi: 10.1080/07357900701805678 18568772

[65] Lee SY Lee KP Lim JW. Identification and biosynthesis of fibrinogen in human uterine cervix carcinoma cells. Thromb Haemost (1996) 75(3):466–70. doi: 10.1055/s-0038-1650298 8701409

[66] Mari-Alexandre J Sanchez-Izquierdo D Gilabert-Estelles J Barcelo-Molina M Braza-Boils A Sandoval J. miRNAs regulation and its role as biomarkers in endometriosis. Int J Mol Sci (2016) 17(1):93. doi: 10.3390/ijms17010093 26771608 PMC4730335

[67] Mari-Alexandre J Barcelo-Molina M Belmonte-Lopez E Garcia-Oms J Estelles A Braza-Boils A. Micro-RNA profile and proteins in peritoneal fluid from women with endometriosis: their relationship with sterility. Fertil Steril (2018) 109(4):675–684 e2. doi: 10.1016/j.fertnstert.2017.11.036 29605406

[68] Sultana S Kajihara T Mizuno Y Sato T Oguro T Kimura M. Overexpression of microRNA-542-3p attenuates the differentiating capacity of endometriotic stromal cells. Reprod Med Biol (2017) 16(2):170–8. doi: 10.1002/rmb2.12028 PMC5661816 29259466

[69] Cai H Zhu XX Li ZF Zhu YP Lang JH. MicroRNA dysregulation and steroid hormone receptor expression in uterine tissues of rats with endometriosis during the implantation window. Chin Med J (Engl) (2018) 131(18):2193–204. doi: 10.4103/0366-6999.240808 PMC6144856 30203794

[70] Braza-Boils A Salloum-Asfar S Mari-Alexandre J Arroyo AB Gonzalez-Conejero R Barcelo-Molina M. Peritoneal fluid modifies the microRNA expression profile in endometrial and endometriotic cells from women with endometriosis. Hum Reprod (2015) 30(10):2292–302. doi: 10.1093/humrep/dev204 26307093

[71] Ujvari D Jakson I Oldmark C Attarha S Alkasalias T Salamon D. Prokineticin 1 is up-regulated by insulin in decidualizing human endometrial stromal cells. J Cell Mol Med (2018) 22(1):163–72. doi: 10.1111/jcmm.13305 PMC5742737 28782224

[72] Naqvi H Mamillapalli R Krikun G Taylor HS. Endometriosis located proximal to or remote from the uterus differentially affects uterine gene expression. Reprod Sci (2016) 23(2):186–91. doi: 10.1177/1933719115613449 PMC5933175 26516123

