# Supplementary figures and images for: Identification of molecular subtypes and immune infiltration in endometriosis: a novel bioinformatics analysis and In vitro validation
Source: Front Immunol. 2023 Aug 18;14:1130738. doi: 10.3389/fimmu.2023.1130738 (PMC10471803; doi:10.3389/fimmu.2023.1130738)

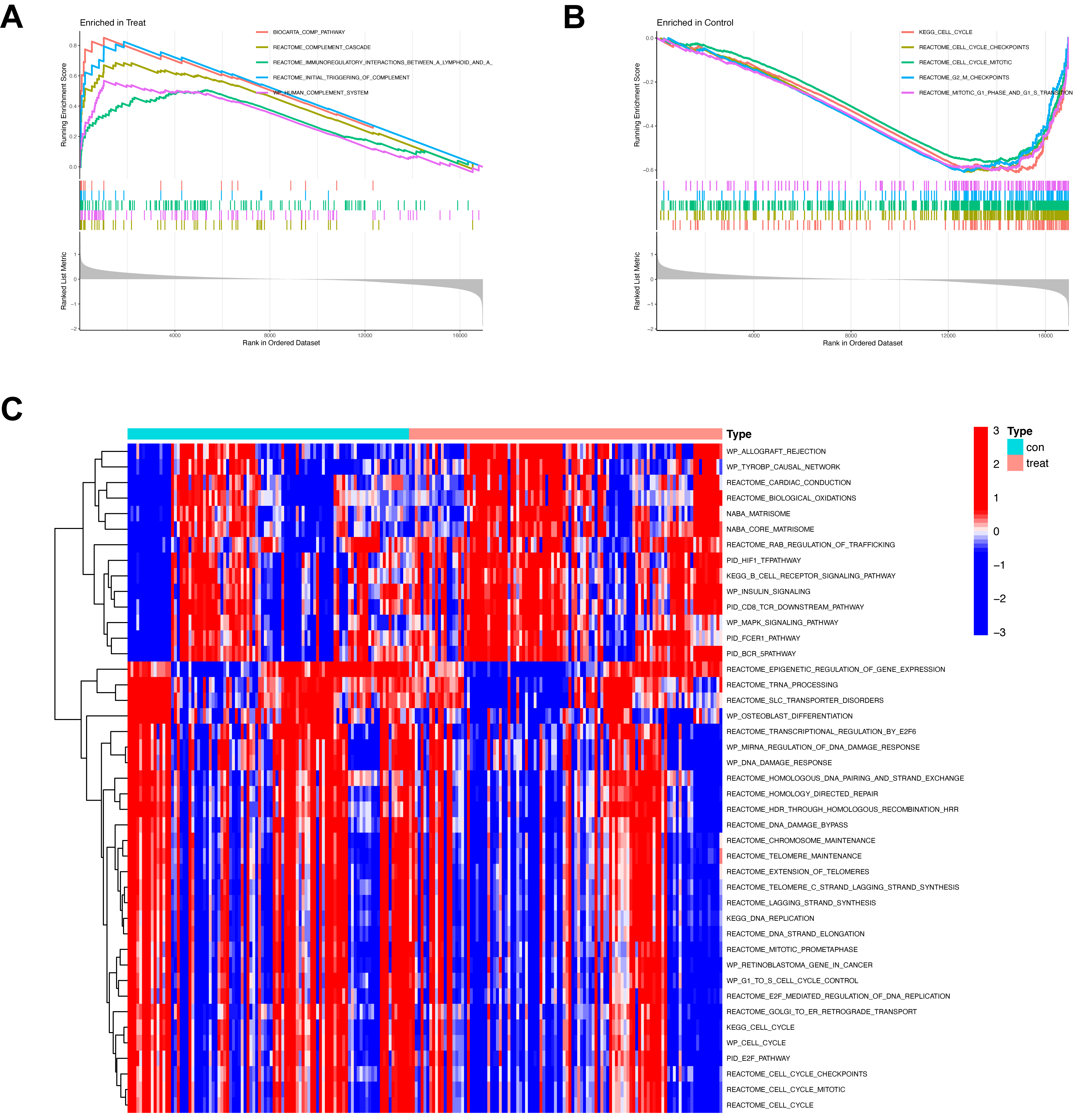

Supplement: Supplementary Figure 1 — Pathways enrichment of DEGs in endometriosis. (A) GSEA results in the endometriosis group. (B) GSEA results in the control group. (C) Heatmap of the enriched pathways. DEGs, differentially expressed genes; GSEA, gene set enrichment analysis. [file Image_1.tif]

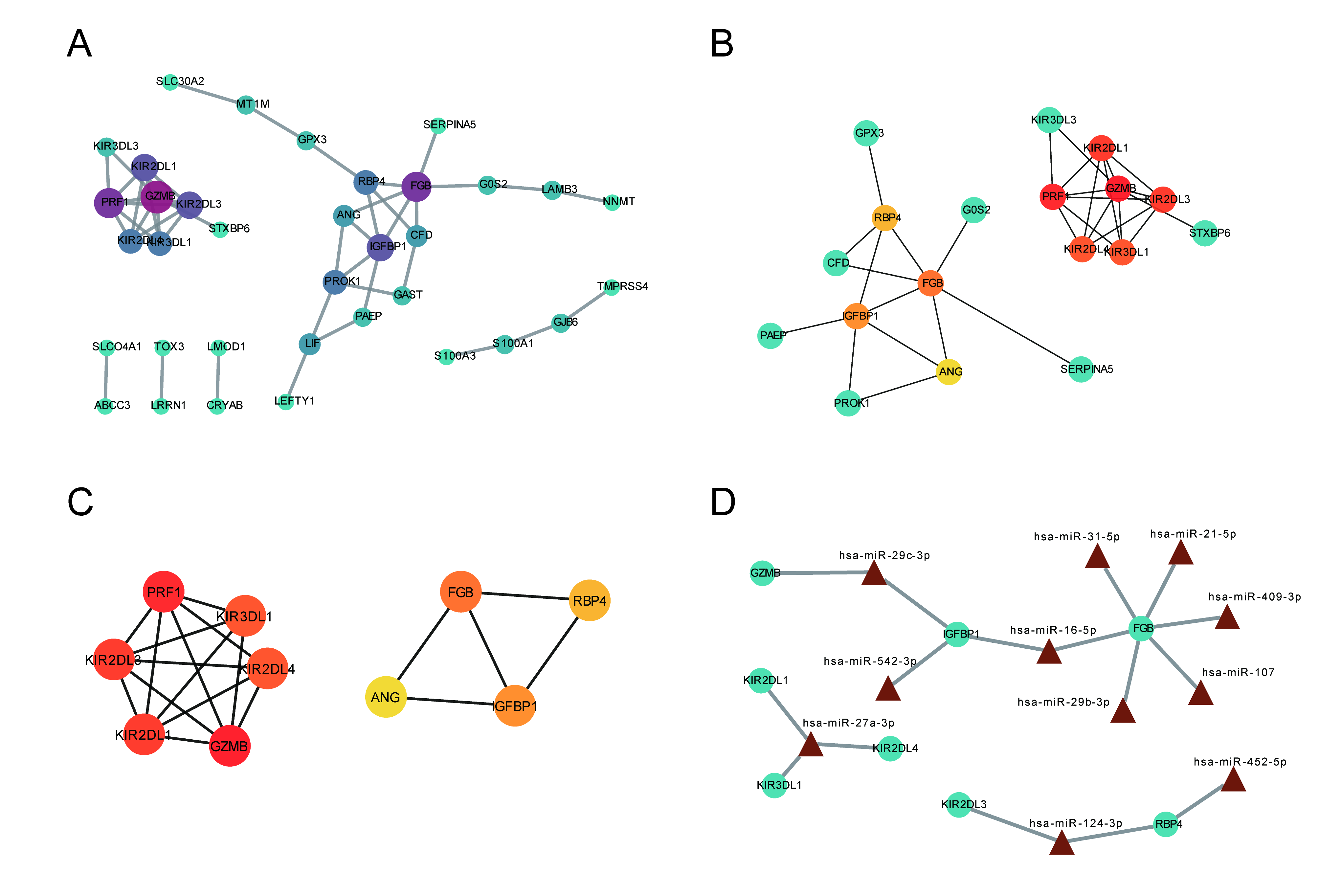

Supplement: Supplementary Figure 2 — PPI network and miRNA-mRNA network analysis. (A) PPI network of 45 common genes constructed by the STRING database. The higher the degree value of the genes, the darker the color and the larger the diameter. (B) Top 10 hub genes and their extensions calculated by cytoHubba. Extension genes were green. The higher enrichment scores were indicated by darker colors. (C) The top 10 hub genes calculated by cytoHubba. The higher enrichment scores were indicated by darker colors. (D) miRNA-mRNA interaction network. Green circles represent mRNA and brown triangles represent miRNAs. PPI, Protein-Protein Interaction. [file Image_2.tif]

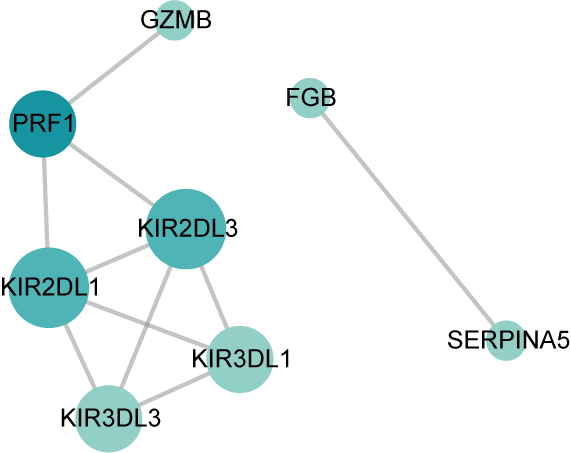

Supplement: Supplementary Figure 3 — PPI network with an interaction score of 0.7. PPI: Protein-Protein Interaction. [file Image_3.tif]

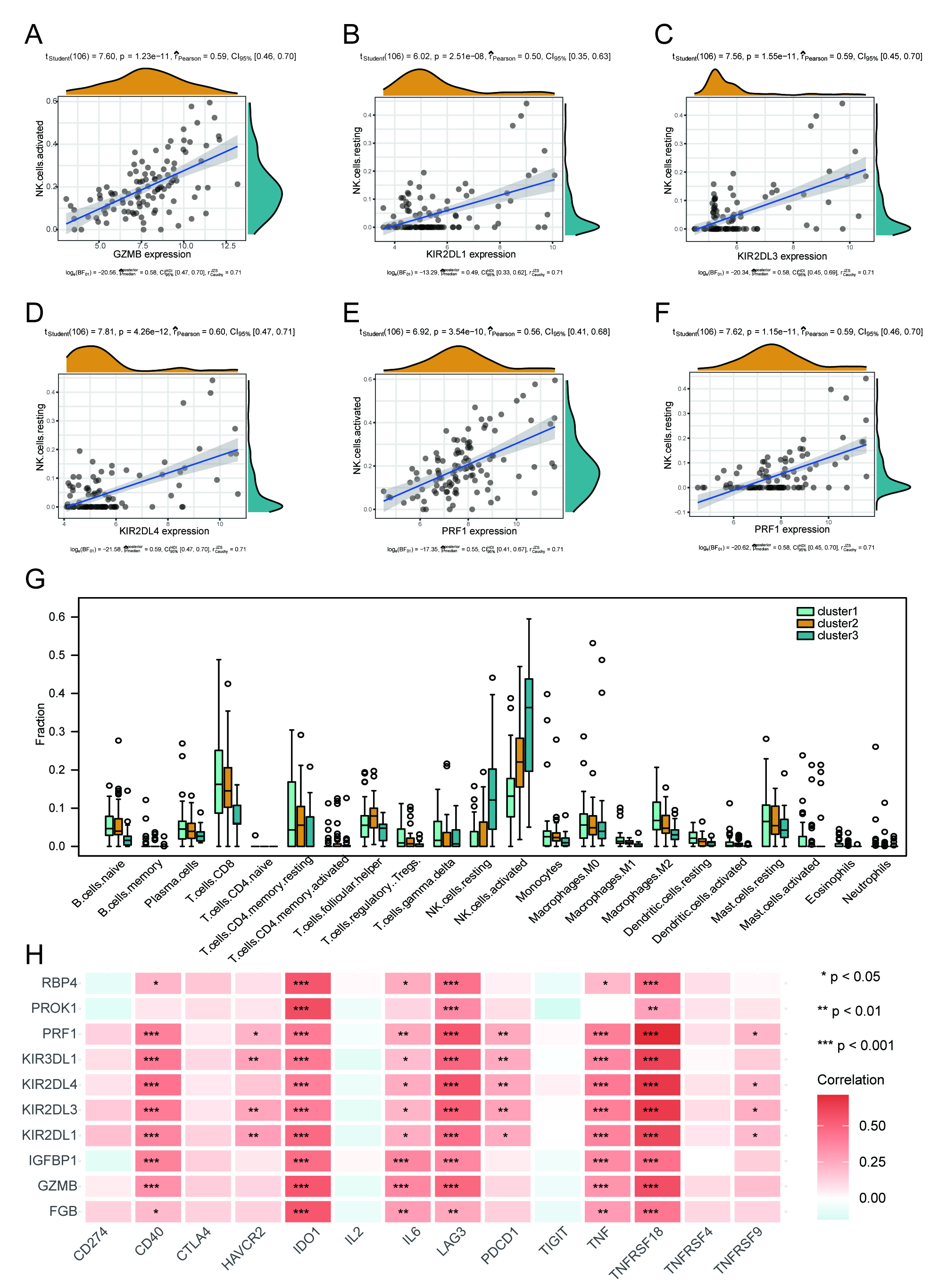

Supplement: Supplementary Figure 4 — Correlation analysis of key genes, immune cell infiltration level, and molecular subtypes. (A) Correlation point plot between GZMB and NK.cells.activated (P = 1.23e-11, R = 0.59). (B) Correlation point plot between KIR2DL1 and NK.cells.resting (P = 2.51e-8, R = 0.50). (C) Correlation point plot between KIR2DL3 and NK.cells.resting (P = 1.55e-11, R = 0.59). (D) Correlation point plot between KIR2DL4 and NK.cells.resting (P = 4.26e-12, R = 0.60). (E) Correlation point plot between PRF1 and NK.cells.activated (P = 3.54e-10, R = 0.56). (F) Correlation point plot between PRF1 and NK.cells.resting (P = 1.15e-11, R = 0.59). (G) Correlation analysis of 22 immune cell fractions in 3 disease subtypes. (H) Correlation between hub genes and immune checkpoint genes. [file Image_4.tif]
