# Supplementary material for: Identification of molecular subtypes and immune infiltration in endometriosis: a novel bioinformatics analysis and In vitro validation
Source: Front Immunol. 2023 Aug 18;14:1130738. doi: 10.3389/fimmu.2023.1130738 (PMC10471803; doi:10.3389/fimmu.2023.1130738)
Supplement: Supplementary Table 4 — GO analysis of central genes. [file Table_4.docx]

| Category | Pathway ID | Pathway description | Count | pvalue |
| --- | --- | --- | --- | --- |
| BP | GO:0001909 | leukocyte mediated cytotoxicity | 4 | 1.63E-04 |
| BP | GO:0045926 | negative regulation of growth | 5 | 4.35E-04 |
| BP | GO:0042267 | natural killer cell mediated cytotoxicity | 3 | 4.92E-04 |
| BP | GO:0002228 | natural killer cell mediated immunity | 3 | 5.95E-04 |
| BP | GO:0051222 | positive regulation of protein transport | 6 | 8.79E-04 |
| BP | GO:0001906 | cell killing | 4 | 9.04E-04 |
| BP | GO:1904951 | positive regulation of establishment of protein localization | 6 | 1.06E-03 |
| BP | GO:0007568 | aging | 5 | 1.36E-03 |
| BP | GO:1900739 | regulation of protein insertion into mitochondrial membrane involved in apoptotic signaling pathway | 2 | 2.02E-03 |
| BP | GO:1900740 | positive regulation of protein insertion into mitochondrial membrane involved in apoptotic signaling pathway | 2 | 2.02E-03 |

**Supplementary Table 4** GO analysis of central genes
