# Supplementary material for: Identification of molecular subtypes and immune infiltration in endometriosis: a novel bioinformatics analysis and In vitro validation
Source: Front Immunol. 2023 Aug 18;14:1130738. doi: 10.3389/fimmu.2023.1130738 (PMC10471803; doi:10.3389/fimmu.2023.1130738)
Supplement: Supplementary Table 5 — KEGG analysis of central genes. [file Table_5.docx]

**Supplementary Table 5**  KEGG analysis of central genes

| ID | Description | Count | pvalue |
| --- | --- | --- | --- |
| hsa05332 | Graft-versus-host disease | 6 | 4.61E-09 |
| hsa04650 | Natural killer cell mediated cytotoxicity | 6 | 4.46E-06 |
| hsa04612 | Antigen processing and presentation | 5 | 5.98E-06 |
| hsa04610 | Complement and coagulation cascades | 3 | 3.01E-03 |
| hsa05330 | Allograft rejection | 2 | 7.49E-03 |
